# Laying a Community-Based Foundation for Data-Driven Semantic Standards in Environmental Health Sciences

**DOI:** 10.1289/ehp.1510438

**Published:** 2016-02-12

**Authors:** Carolyn J. Mattingly, Rebecca Boyles, Cindy P. Lawler, Astrid C. Haugen, Allen Dearry, Melissa Haendel

**Affiliations:** 1 Department of Biological Sciences, and; 2 Center for Human Health and the Environment, North Carolina State University, Raleigh, North Carolina, USA; 3 National Institute of Environmental Health Sciences, National Institutes of Health, Department of Health and Human Services, Research Triangle Park, North Carolina, USA; 4 Library, and; 5 Department of Medical Informatics and Clinical Epidemiology, Oregon Health & Science University, Portland, Oregon, USA

## Abstract

**Background:**

Despite increasing availability of environmental health science (EHS) data, development, and implementation of relevant semantic standards, such as ontologies or hierarchical vocabularies, has lagged. Consequently, integration and analysis of information needed to better model environmental influences on human health remains a significant challenge.

**Objectives:**

We aimed to identify a committed community and mechanisms needed to develop EHS semantic standards that will advance understanding about the impacts of environmental exposures on human disease.

**Methods:**

The National Institute of Environmental Health Sciences sponsored the “Workshop for the Development of a Framework for Environmental Health Science Language” hosted at North Carolina State University on 15–16 September 2014. Through the assembly of data generators, users, publishers, and funders, we aimed to develop a foundation for enabling the development of community-based and data-driven standards that will ultimately improve standardization, sharing, and interoperability of EHS information.

**Discussion:**

Creating and maintaining an EHS common language is a continuous and iterative process, requiring community building around research interests and needs, enabling integration and reuse of existing data, and providing a low barrier of access for researchers needing to use or extend such a resource.

**Conclusions:**

Recommendations included developing a community-supported web-based toolkit that would enable a) collaborative development of EHS research questions and use cases, b) construction of user-friendly tools for searching and extending existing semantic resources, c) education and guidance about standards and their implementation, and d) creation of a plan for governance and sustainability.

**Citation:**

Mattingly CJ, Boyles R, Lawler CP, Haugen AC, Dearry A, Haendel M. 2016. Laying a community-based foundation for data-driven semantic standards in environmental health sciences. Environ Health Perspect 124:1136–1140; http://dx.doi.org/10.1289/ehp.1510438

## Introduction

This review is derived from a workshop held at North Carolina State University, Raleigh, North Carolina, USA, on 15–16 September 2014. Sharing, analysis and integration of environmental health science (EHS) data is limited by a lack of data standards, in particular, common language standards. Language standards are shared vocabularies that are used for data annotation and common data elements specification to aid interoperability. They may be as complex as an ontology, whereby the terms and the relations between them are defined using logic and are expressed in computable languages such as the Web Ontology Language (OWL 2016), or, they may be as simple as a hierarchical vocabulary. This workshop aimed to *a*) articulate research areas that would be advanced by EHS language standards and data interoperability, *b*) identify a community to initiate the creation and champion the extension of EHS language standards, and *c*) develop guidelines for the development of EHS standards.

Exposure to environmental factors significantly impacts human health. The environment, broadly defined, can range from everyday products (e.g., toothpaste) to hazardous materials (e.g., open pit mining sites) and socioeconomic stressors. Consideration of this spectrum is needed to better understand how, when, and to whom exposures pose health risks. There is an enormity of available data that, if structured and integrated, could be leveraged to inform mechanistic hypotheses, therapeutic approaches, and policy making. However, a lack of semantic standards has been a major barrier to data sharing and integration (van Panhuis et al. 2014). This need for semantic standards is being recognized in many areas of biomedical research. For example, the National Research Council’s report titled “Toward Precision Medicine” called for clinical and research advancements based upon systems that would be enabled by a new language standard (NRC 2011). The authors of this report—Committee on a Framework for Developing a New Taxonomy of Disease, Board on Life Sciences, and Division on Earth and Life Studies—determined that “The rise of data-intensive biology, advances in information technology, and changes in the way health care is delivered have created a compelling opportunity to improve the diagnosis and treatment of disease by developing a Knowledge Network, and associated New Taxonomy, that would integrate biological, patient, and outcomes data on a scale hitherto beyond our reach” (NRC 2011).

Development of semantic standards, such as logically constructed ontologies, EHS data, and integration of this effort within the broader biomedical context through crosscutting research programs, such as the Exposome (Wild 2005) and Big Data to Knowledge (BD2K) (Margolis et al. 2014), will enhance the capacity to inform disease research with environmental data while also improving understanding of environmental impacts on human disease. The lack of language standards and their consistent implementation affects not only the capacity to analyze across diverse data sets, but even hinders the ability to identify available data sets, limiting the value of potentially important scientific findings. A query of microbiome samples using PubMed from the National Center for Biotechnology Information (NCBI 2016) illustrates the variability in results that stem from a lack of harmonized language standards and annotation of data using such standards (Table 1). Standardization has the potential to benefit many areas of biomedical science by augmenting discovery and reuse (Richesson and Nadkarni 2011; Tenopir et al. 2015; Zimmerman 2008).

**Table 1:** Variable results from a PubMed query of microbiome samples illustrates the consequences of lacking semantic standards and implementation (NCBI 2016).

Query	No. of results
Feces	22,592
Faeces	1,750
Ordure	2
Dung	19
Manure	154
Excreta	153
Stool	22,756
Stool NOT faeces	21,798
Stool NOT feces	18,314

A few projects have specifically demonstrated the potential of adopting standards to advance EHS data integration, research, and discovery. For example, the Oceans and Human Health program [supported by the National Institute of Environmental Health Sciences (NIEHS) and the National Science Foundation] links oceanographic and metagenomics data sets (NCBI’s Sequence Read Archive, Metagenomic Rapid Annotations using Subsystems Technology) (NCBI-SRA 2015; Youngblood et al. 2014), and custom public health databases (Antibiotic Resistance Database, Computer Access to Research on Dietary Supplements Database) (Liu and Pop 2009; NIH 2015) using ontologies to provide an innovative, health-based framing for oceanographic observatories (microbial diversity and antibiotic resistance of ocean ecosystems) (Port et al. 2012, 2014). The Comparative Toxicogenomics Database (CTD) (Davis et al. 2015) provides integrated information about chemicals, genes and proteins, phenotypes, diseases, and exposures to provide mechanistic insights into the effects of the environment on human health (Davis et al. 2015). Data are annotated and integrated using public ontologies describing chemicals (MeSH) (NLM 2015b), genes and proteins (Entrez Gene) (NLM 2015a), diseases (MeSH), and interactions (CTD interaction ontology) (Davis et al. 2015). Consequently, users may query cross-species mechanistic data for specific or broad classes of chemicals and identify associated diseases or disease models. Broader development and adoption of EHS standards will be necessary to ensure access, re-use, innovative integration, and ongoing re-analysis of data that describe the complex interactions between the environment and human health.

## Discussion

### Gaps in EHS Semantic Standards

The data standardization needs within EHS are diverse and include genomics, metabolomics, chemistry, toxicology, epidemiology, exposure science, phenotypes, geospatial data, and clinical health records among others. While some of these components are better standardized than others (e.g., genomics) and not necessarily specific to EHS, it is the need for integration across these diverse entities in order to better model the complexity of environmental health interactions that is unique. In apparent contradiction, there are a large number of existing standards (Tenenbaum et al. 2014), yet often the needed content is missing, occurs redundantly in more than one context, or cannot be found. Although there are several public resources that have centralized some publicly available semantic vocabulary standards and ontologies (OboFoundry, NCBO BioPortal, Biosharing.org, Ontobee) (Biosharing 2015; NCBO 2015; Smith et al. 2007; Xiang et al. 2011), there is still limited capacity for the community to identify the concepts they need across the spectrum, contribute in such a way that reduces redundancy and enhances existing standards, and easily compare the content between selected standards. In addition, few of these resources are associated with the data that are annotated using the ontologies or vocabularies. This disconnect leads to semantic standards that are not necessarily built fit-for-purpose and lack examples that would help users determine which standards would be most appropriate for their needs. There is a need for a tool in this space to inform decision making about the incorporation of an existing standard, the need to extend such resources, or create and coordinate new standards. Critical to this decision making is the need to link to existing data sets in which semantic standards have been applied and understand the impacts of standards use and evolution on downstream data analyses. Further, EHS needs to incorporate emerging biomedical concepts (e.g., the exposome) that are not adequately represented among existing vocabulary resources. Consequently, there is a need for tools that allow community-based development of new standards, such as in cases of emerging research areas.

A critical component of development and adoption of semantic standards is community agreement on the meaning of terms and their use in different contexts. Gaining agreement is often difficult and imperfect, and consideration should be given to achieving agreement where there is a natural propensity, whether at a specific level of detail or around specific concepts. Semantic disagreements can be due to community diversity, overspecification of terms, or changes in the meaning of terms over time. In cases where agreement cannot be achieved, community-specific synonyms must be incorporated to avoid limiting the utility of the standard or stalling future development. Furthermore, once a standard is available, its value is largely determined by the datasets and projects that adopt it. Wide adoption of standards is best achieved when diverse constituencies, such as data generators, data users, standards developers, publishers, and government agencies are involved and incentivized to participate in community education, participation, and tool building. New tools are needed to cultivate a greater degree of collaborative development.

### Lessons Learned

The Gene Ontology (GO) (Ashburner et al. 2000) is often referenced as a gold standard for ontology-based initiatives by virtue of its global community participation and implementation, development of tools to browse and access content, and its impact on data integration and analysis; however, it had humble beginnings, and there is much to be learned from its early roots and subsequent path. Developed with input from an international consortium to represent how genes encode biological functions at the molecular, cellular, and tissue system levels across diverse species, GO now describes more than 40,000 biological concepts (GO 2015). GO annotations are incorporated into countless biological resources and it has been cited in over 100,000 peer-reviewed articles (GO 2015). GO has enabled integrative analyses that are now common in genomic experiments, such as gene set enrichment. Drawing upon GO, the following successful features of a process for developing semantic standards were identified:

Start with simple and practical initiatives.
Utilize a modular building block approach to allow for flexibility and reuse.
Leverage and interoperate with existing standards where possible.
Develop language standards to work with scientific uncertainty.
Find balance between logic engineering and easier-to-use vocabulary editing.
Develop standards in close contact with the data and specific scientific need.
Focus on capturing scientific findings (i.e., durable facts).
Facilitate community-based collaborative curation of term definition and annotation.
Provide stable unique identifiers.
Incorporate significant time for community engagement and debate.
Provide accessible user interfaces for ongoing development.

### Guiding Principles

In order to ensure buy-in and use of EHS standards, we provide the following eight recommendations for establishing a community willing to participate in the development of an EHS ontology and the resources needed to accomplish this development.

***Engage a broad community*.** Consider a broad community of stakeholders including researchers and clinicians (data generators and data users), publishers, and government agencies. Engagement can be achieved through stand-alone workshops, events that are embedded within broader yet aligned programs [e.g., Big Data to Knowledge (BD2K)] (Margolis et al. 2014), and web-based resources where users can participate in discussions or add to data sets.

***Facilitate collaboration*.** Proactively enable collaborations by planning for redundancy or inconsistency across terms within standard resources. A web-based, automated method of identifying incongruences between concepts would provide the user a valuable status check across specified terms. By highlighting these inconsistencies, they may be collaboratively resolved.

***Enable navigation of existing language standards*.** Current inventories of standards resources lack descriptive details about the standards themselves as well as applications for which they have been used. There is a need for details that allow a user an accessible glimpse of what “coverage” exists, perhaps by terms or functions of standards, as well as how they have been used to standardize data. The EHS research community should be able to easily find and evaluate standards for use with their own data.

***Support citation and attribution of semantic standards*.** A language standard used within a project, data resource, report, publication, or other scholarly product needs to be a citable entity. Standards contributions must be recognized scholarly endeavors to incentivize development. Small contributions to languages (e.g., creation of classes in ontologies) can be tracked with contributor identifiers (IDs) (e.g., ORCID IDs) (ORCID 2015). Attribution to funding entities (e.g., grant awards) may also be included to fully capture the roles within the standard lifecycle.

***Adopt software development best practices*.** Development should address a need in the context of real data. Break the work into modularized portions and provide descriptors for each module. Utilize robust version control and attribution for each module as routinely practiced in software development. Publish each module to enable testing, reuse, and integration by others.

***Assist early development*.** One challenge is that the early stages of standards development are rarely funded, but the initial stages of projects involving standards are crucial for establishing effective collaborations. Small funding sources for collaborative exchanges can help. The National Science Foundation Research Coordination Networks (RCN) (NSF 2015) is one mechanism for this, but there could be a more general funding mechanism.

***Be sustainable and flexible*.** A successful framework must allow for continuous iteration of standards, be extensible in the face of evolving technologies, be driven by the data and community needs, and enable community participation and crowdsourcing.

***Capitalize on opportunistic development*.** Seek existing projects or use cases that require or are developing language standards. Utilize these opportunities to pilot a framework approach.

These guiding principles should be operationalized to serve as a resource for the EHS research community. A web-based toolkit could enable navigation of relevant standards from existing sources and serve as a collaborative infrastructure for community-based participatory research. Such a resource could include navigation not only of existing standards, but also the data within resources that leverage those standards. This connection would facilitate crowdsourcing approaches and tool development such as trackers, forum pages for the community to contribute use cases, and success stories. The intention of such a toolkit would be to complement and work synergistically to achieve an environmental health slice of existing standards efforts and technologies. For example, a project investigating the microbiome population and its response to different dietary and environmental exposures needed to standardize *a*) the microbiome species, *b*) the source from which the microbiome sample was taken (e.g., stool, mouth, etc.), *c*) a set of key nutrients, *d*) environmental contaminants, and *e*) disease and phenotypic characteristics at the time of sampling. The EHS toolkit could potentially go to the Human Microbiome Project (NIH HMP Working Group et al. 2009) to uniquely identify microbial strains, collect anatomical terms from the Uberon anatomy ontology (Haendel et al. 2014), foods from Wikipedia (2015), target chemicals from MeSH (NLM 2015b), diseases from the Disease Ontology (Schriml and Mitraka 2015), and phenotypes from the Human Phenotype Ontology project (Köhler et al. 2014). In choosing the terms, the user would want to see what data were already associated—for example, which phenotypes had been associated with the candidate disease? Which toxicants were found in the groundwater near certain population(s)? The output would be a logically constructed collection of vocabulary terms that could be used in the project, edited, and contributed back to the source resources, while maintaining provenance.

Development of an EHS toolkit would require expertise in technical standards development processes, such as software engineering that leverages the Web Ontology Language (OWL 2016). It would also require close collaboration with the various sources of vocabulary standards to support interoperability and coordination of community contributions, and environmental health related data resource developers. Finally, tools such as Web Protégé (WebProtege 2015) or Semantic Media Wiki (SMW 2015), if enhanced with functionality to meet the above needs, may potentially be utilized as web-based locations for collaborative editing, reviewing, and sharing the slices of the vocabulary standards.

### Phased Approach to EHS Semantic Standards Development

There are several current challenges to development and broad adoption of EHS semantic standards including identification of an invested community, accessibility of semantic standards and development resources, and availability of funding to ensure ongoing support and sustainability. A major accomplishment of this workshop was identification of a community, composed of the workshop participants, who are committed to initiating and participating in a collaborative effort to develop EHS semantic standards. This community strongly recommended *a*) federal funding to ensure augmentation and adoption of these standards and *b*) interdependent and iterative phases of development described below.

***Phase I: Identify EHS research questions and use cases.*** To ensure currency and immediate application, the EHS community should focus semantic development efforts related to current research questions. Refinement through development of detailed use cases within the community forum would serve to engage the multidisciplinary EHS community and help to prioritize standards development: use cases to minimally describe a research question; the data, standards, and resources required to address the question including gaps in knowledge; and competency questions (essentially questions that are used to test adequacy of the standard) that enable clear and focused communication around the research need. To facilitate progress, we developed a use case template and applied it to a sample research question (see Supplemental Material, “Use Case Template”). Initial research questions and use cases should attempt to use existing or ongoing data input streams. By embracing a needs-based approach and working openly to provide solutions on a focused, well-understood project, development efforts are more likely to evolve and address real research needs. Currently, through a listserv mechanism (see below), our community has begun the process of identifying research areas for use case development. Development of use cases would be an ongoing activity that serves to expand EHS data representation and the capacity for integration and reuse over time.

**Figure 1:**
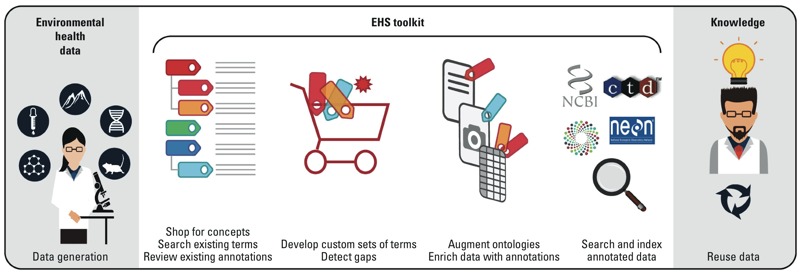
An EHS Semantic Toolkit (Phase II). We recommend establishing a web-based toolkit to facilitate exchange, extension, and adoption of EHS data standards. Priority areas of research and associated use cases (Phase I) will drive the use of the toolkit, which will allow users to *a*) search broadly for EHS concepts and related existing terms and evaluate the context of terms through associated annotations in knowledge bases; *b*) develop custom sets of terms to address their use cases and detect gaps in available standards; *c*) extend existing ontologies and enrich new terms with associated annotations; and *d*) continually expand the search capability of identifying and reusing data standards. This workflow will inform the development of a governance and sustainability plan to ensure ongoing use and expansion in increasingly broader and cross-disciplinary contexts (Phase III). This cycle will iterate as more research questions are identified and the community becomes more involved.

***Phase II: Develop a web-based, EHS standards toolkit.*** We propose development of a web-based toolkit that will support navigation of existing standards, knowledge, and data sources; and allow users to extract vocabulary slices for a given research project and enable extension of these standards (Figure 1). The overall goal is to provide *a*) navigation of environmental health relevant vocabulary standards and concepts that can be found in a broad diversity of locations on the web; *b*) allow custom term set creation (slices) in a logically consistent, shareable, and reusable manner; *c*) allow perusal of existing data to inform term selection and enable quality assurance; and *d*) provide a brokering mechanism to contribute terms and edits back to the source vocabularies and knowledgebases. This tool would therefore facilitate crowdsourcing vocabulary development. Group sharing of the slices could potentially increase the EHS community’s adoption and extension of existing standards, and provide a mechanism for broadening the collection of research questions, use cases, and success stories described in Phase 1.

To facilitate participation, data entry and automated validation tools for quality control assessment were recommended as part of the toolkit. One example of a validation tool is the Annotation Sufficiency Meter (Phenoday 2014) provided by the Monarch Initiative (Monarch 2015), which leverages diverse large-scale semantically integrated data. This validation tool allows clinicians or model organism researchers to enter phenotypic data at the point of care or in the lab, and then get back quality assurance metrics on their phenotype ontology annotations. It will be critical for those experienced in developing such resources to help develop tools that leverage language standards and data stores together. This integration will ensure that researchers benefit during the process of data creation, analysis, and publication from the use of language standards while simultaneously contributing to them.

***Phase III: Develop a plan for governance and sustainability.*** A governance model is essential for maintaining a coordinated suite of semantic standards, sustaining community efforts in keeping with scientific and technical advances, and championing public availability of standards for EHS data to ensure continued relevancy. Governance should involve representation from the full spectrum of data-generating labs, funders, domain scientists, informaticians, and publicly available resources. Modern open source software development environments, such as GitHub (2015), have become more accessible to the layperson and have been extremely successful in coordinating distributed vocabulary development projects. We recommend coordinating with such efforts as well as emerging funding mechanisms (e.g., BD2K, Children’s Health Exposure Analysis Resource, and other exposome initiatives at NIEHS) (NIH 2016; Margolis et al. 2014) for which standards development is an expressed need in the interest of establishing best practices and avoiding redundancy.

A common problem for resource development projects such as databases or ontologies is the lack of dedicated and sustainable funding mechanisms. A paradigm shift by funding agencies and reviewers is needed such that development of data resources is not evaluated through the same lens of traditional hypothesis-driven research projects. Effective and broadly used semantic standards require a high level of scholarship and community involvement, result in major capacity-building impacts on research, and are increasingly recognized for their integral role in data analysis and integration; yet there are virtually no dedicated funding mechanisms for their development or sustainability. Dedicated funding mechanisms are needed as stand-alone or as ongoing research programs. For either mechanism, funding agencies should consider in advance how developed resources will be sustained long term and integrated into other ongoing research projects. To justify continued funding, metrics that reflect scientific value must be incorporated to track use (e.g., numbers of citations where semantic resources were used). Although seemingly straightforward, such metrics are challenging to compile because infrastructure and standards are generally not well cited, web-based tracking is not uniformly defined and can be wildly misleading, and new metrics are needed to properly credit infrastructure developers and collaborative teams that are not based solely on publications (NIH 2014). Many of these issues are not unique to EHS; however, the lack of semantic standards for EHS-specific areas (e.g., exposure-related contexts, chemicals) and the need for improved integration within the broader biomedical research landscape will only be rectified by the EHS community and associated funding.

## Conclusions

It is an opportune time for the EHS community to help catalyze the development of standards given the increasing quantity and diversity of data that is poised to advance our understanding about environmental impacts on human health. Lessons from previous language standard development efforts emphasize the long-term nature of such endeavors, and that persistence and endurance are critical characteristics of successful efforts. Toward this end, sustaining community engagement is critical, and a phased approach is recommended: *a*) develop EHS research questions and use cases; *b*) identify existing language resources and build navigational tools to encourage adoption and extension; and *c*) determine a plan for governance and sustainability.

Clearly such advances will require dedication of resources, must address real needs, remain close to the data, and follow a sustained, but phased approach. In the coming months, NIEHS will pursue an engagement and outreach strategy providing a listserv for discussion and dissemination of materials, a research question and use case template, and a sample semantic standard inventory to be used in a community forum to give shape to the recommendations that have been described in this report. To contribute to this community, please register with the listserv at EHSCOMMONLANGUAGE@LIST.NIH.GOV.

## Supplemental Material

(179 KB) PDF
