# Supplementary material for: Laying a Community-Based Foundation for Data-Driven Semantic Standards in Environmental Health Sciences
Source: Environ Health Perspect. 2016 Feb 12;124(8):1136–40. doi: 10.1289/ehp.1510438 (PMC4977056; doi:10.1289/ehp.1510438)
Supplement: (179 KB) PDF [file ehp.1510438.s001.acco.pdf]

Environ Health Perspect
DOI: 10.1289/ehp.1510438

Note to readers with disabilities: *EHP* strives to ensure that all journal content is accessible to all readers. However, some figures and Supplemental Material published in *EHP* articles may not conform to 508 standards due to the complexity of the information being presented. If you need assistance accessing journal content, please contact ehp508@niehs.nih.gov. Our staff will work with you to assess and meet your accessibility needs within 3 working days.

Supplemental Material

Laying a Community-Based Foundation for Data-Driven Semantic Standards in Environmental Health Sciences

Carolyn J. Mattingly, Rebecca Boyles, Cindy P. Lawler, Astrid C. Haugen, Allen Dearry, and Melissa Haendel

Table of Contents

Use Case Template
- ***A. Scientific question, premise, or problem statement***
- ***B. Data sources and types***
- ***C. Competency or requirements questions***

Use Case Example from the Workshop: Integration of CTD (CTD 2015; Davis et al. 2014) and Monarch (Monarch 2015) Data to Build Chemical-Gene-Phenotype Relationships
- ***A. Scientific problem statement***
- ***B. Data sources and types***
- ***C. Competency or requirements questions***

Here we provide a template to assist community members in developing use cases that can best support requirements analysis. The use case necessarily spans broad biological issues, and then narrows down to the specific data, models, and test (competency) questions that are used to evaluate the vocabularies and models to determine whether they meet the need of the broader issues.

Use Case Template

A. Scientific question, premise, or problem statement. Here one must pose some kind of scientific inquiry. This initial scoping can be broad or narrow. A narrow example could be “I would like to understand the correlation of Lithium in the groundwater with psychiatric outcomes of patients with bipolar disorder family history.” A broader example might be “I would like to understand the effects of diet on diseases of the liver.” General goals or problem statements can be listed here, such as: “drug treatment of psychiatric illness is confounded by environmental variables such as groundwater chemicals and diet, these need to be taken into account during treatment decisions.” Use cases can be biological in nature, social, or technical.

B. Data sources and types. Include the following details for each data source that will be leveraged to address the above problem statement. Reference use of any existing data standards/formats or vocabularies/ontologies.

1. **Source**: Person or organization that “owns” the data (include link and/or citation)
    a. **Description**: Describe the nature of the study/project that generated the data (e.g., Patient study data).
    b. **Type**: Describe what the data represents (e.g., Dietary preferences).
    c. **Format/data details**: Describe the format of the data and use of any standardized identifiers, vocabularies, or other data standards (e.g., Diet items are recorded using a

self-defined controlled vocabulary using RedCap (RedCap 2016); the amounts are recorded as times per week).

d. **Sample data**: Here you would include for each source/type listed above, a row or example of the data. Highlight components that conform to standards.

e. **Gap(s)**: List potential data gaps or limitations to the data set. If the missing data is essential, provide a comment about how this limitation will be addressed.

Note that for sources with multiple Types, each Type should be listed with its own format/data details, sample data and gaps unless this information is consistent for all Types.

C. Competency or requirements questions. Provide questions that will be enabled by integration and alignment of the above data. Questions should range from very simple to more complex. They will be used to design the data integration structure and determine downstream functionality. This list can be short at first, but lists of questions over a hundred are not uncommon when designing a semantic structure to be utilized within a search system. These questions are analyzed for semantic components and data availability/integrity as well as to test system function. Include the source/person that provided the question, as you will return later to see if the results are as they expected. Examples:

1. Search for patients who drink soft drinks.
2. Search for patients with liver inclusion phenotypes and who drink more than 4 drinks/day.
3. Search for diseases that are correlated with mutations in nuclear hormone receptor genes and patients with significant exposure to environmental estrogens.

<u>Use Case Example from the Workshop: Integration of CTD (CTD 2015; Davis et al. 2014) and Monarch (Monarch 2015) Data to Build Chemical-Gene-Phenotype Relationships</u>

A. Scientific problem statement:

Identification of protein targets of environmental chemicals or secondary drug targets is difficult due to sparse data, lack of integration across *in vitro* and *in vivo* models, lack of uniformly correlating genotype to phenotypes and their relationships to environmental variables, and poor temporal representation of exposure conditions and measurements. To support better understanding about mechanisms of chemical actions and inform secondary drug use, we need to be able to query across diverse data sources and types that are currently disconnected. The outcomes will be next steps for experimental validation, *in vivo* model evaluation, and eventually phase I clinical trials.

B. Data sources and types.

1. **Source**: Comparative Toxicogenomics Database
   a. **Data description**: Curated chemical-gene-disease and exposure data from the literature
   b. **Type 1**: Chemical-gene/protein interactions
      i. **Format/data details:** Chemical-gene interactions are captured manually and also include associated species information. Chemicals are annotated using Medical Subject Headings (MeSH) (MeSH 2015) chemicals and drugs vocabulary. Genes and proteins are annotated using the National Center for Biotechnology Information's (NCBI) Entrez Gene vocabulary (Entrez-Gene 2015). Species are annotated using the NCBI Taxonomy vocabulary (Taxonomy 2015). Interactions are characterized using the CTD action vocabulary (Davis et al. 2011); the form of the chemical and gene (e.g., protein, promoter region) can be qualified using internal notation. Degree of the

effect can also be qualified with -, + or 0 when the interaction is decreased, increased or not specified, respectively.

  ii. **Sample data**: Chlorpyrifos results in increased expression of ABCA1A mRNA; CTD Chemical-gene interaction page

c. **Type 2:** Chemical-disease relationships

  i. **Format/data details**: Chemical-disease relationships are captured manually and also include species information. Chemicals are annotated using the MeSH chemicals and drugs vocabulary. Diseases are annotated using the Merged Disease Vocabulary, MEDIC (Davis et al. 2012), which is a mapping of the Online Mendelian Inheritance of Man (OMIM) (OMIM 2015)diseases to the MeSH diseases vocabulary (mapping was done by CTD staff and published)(Davis et al. 2012). Species are annotated using the NCBI Taxonomy vocabulary. Relationships can be qualified as Therapeutic or Marker/Mechanism.

  Species are annotated as described above (Type 1). Relationships can be qualified as Therapeutic or Marker/Mechanism.

  ii. **Sample data**: CTD chemical-disease page

d. **Type 3**: Gene-disease relationships

  i. **Format/data details**: Gene-disease relationships are captured manually and also include species information. Genes and proteins are annotated using the Entrez Gene vocabulary. Diseases and species are annotated as described above. Relationships can be qualified as Therapeutic or Marker/Mechanism.

  ii. **Sample data**: CTD gene-disease page

e. **Gap(s)**: Currently CTD captures diseases related to genes and chemicals. However, there is a need to identify phenotypes that may precede diseases if we are to improve early diagnosis or treatment. In addition, phenotypes at a more basal level than a full-blown disease may facilitate comparisons across model systems. Until recently, CTD lacked such phenotypes at the cellular or organ/tissue level. We have expanded our curation scope to include phenotypes; however, this is a labor-intensive process with many options for capturing phenotype information across species. Monarch (see source 2) currently contains abundant human phenotype data and have developed annotation protocols that will help to inform a coordinated curation and data sharing effort between Monarch and CTD.

2. **Source**: Monarch Initiative

   a. **Data description**: Integrated genotype-phenotype relationships across human and *in vivo* and *in vitro* models of disease. Available are phenotype-to-disease associations using the Human Phenotype ontology (Kohler et al. 2014), the Gene Ontology (Ashburner et al. 2000), and numerous other organismal phenotype ontologies from mouse, rat and zebrafish. Phenotypes use identifiers in the obolibrary (obophenotype 2016) phenotype ontology namespace.

   b. **Type 1:** Gene-disease associations. Available are human Mendelian gene-disease associations, as well as Copy number variant gene-disease associations.

      i. **Format/data details:** Diseases are recorded using a combination of OMIM, Decipher (DECIPHER 2015), and Orphanet (orphanet 2015)vocabulary identifiers using an integrated semantic framework available here. Genes use NCBI Entrez Gene IDs.

ii. **Sample data**: Available here. On the gene tab are shown genes and associated with Parkinson's Disease and its subtypes from a variety of sources as indicated on the right-most column.

c. **Type 2**: Disease-phenotype associations.

i. **Format/data details:** Disease-phenotype associations are captured manually. Diseases are annotated as per above disease semantic framework, and are annotated using the human Phenotype Ontology. Frequency of the phenotypes is recorded, as well as evidence, and age of onset.

ii. **Sample data**: The data as annotated are located here (available on the phenotype tab of any Disease page in Monarch).

d. **Type 3**. *In vitro* models-disease associations. Available are links between cell lines and diseases from different organisms.

i. **Format/data details:** Cell lines are from a variety of sources and are annotated with the aforementioned disease integrated framework. The data can be found in the models tab of any Monarch disease page.

ii. **Sample data:** An example is found here.

Gaps:

Currently Monarch data have not been curated/aligned with exposure/drug vocabularies or identifiers, which could include specific genetic perturbations, local environmental exposures, or epidemiological findings. Clinical/model organism measurements within specific trait areas are different in different organisms but they are used to assess similar traits. These traits need to be integrated with abnormal phenotypes. The Disease integrated semantic framework used in Monarch is not exactly the same as is used in CTD, though both draw on some of the same

sources. Some of the data sources used within CTD are the same as Monarch, and there is overlapping literature curation.

C. ***Competency or requirements questions:***

1. Can integration of cross-species phenotypes, chemical interactions and disease data help to identify phenotypic fingerprints for environmentally influenced diseases?
2. Can we improve identification of candidate targets of exposures by retrieving similar phenotype data across diverse disease models (e.g., zebrafish, mice)?
3. Do candidate chemical targets in in vivo models and in vitro model systems correlate?
4. Can integration of cross-species phenotype data help inform human disease progression?
5. What gene networks are related to a given set of phenotypes associated with an exposure?
6. Can mechanism of action assessments be improved by making phenotypic connections across experimental systems (in vitro, cross-species)?
7. Can overlapping phenotypes and their associated genes help to identify basic underlying mechanisms shared by seemingly unrelated diseases?
8. Can the establishment of chemical-gene-pathway-phenotype-disease continuums help to identify biomarkers of disease onset?

References

Ashburner M, Ball CA, Blake JA, Botstein D, Butler H, Cherry JM, et al. 2000. Gene ontology: tool for the unification of biology. The Gene Ontology Consortium. Nature genetics 25:25-29.

CTD (Comparative Toxicogenomics Database). 2015. Available: http://ctd.mdibl.org [accessed 10 October 2015].

Davis AP, Wiegers TC, Murphy CG, Mattingly CJ. 2011. The curation paradigm and application tool used for manual curation of the scientific literature at the Comparative Toxicogenomics Database. Database (Oxford) 2011:bar034.

Davis AP, Wiegers TC, Rosenstein MC, Mattingly CJ. 2012. MEDIC: a practical disease vocabulary used at the Comparative Toxicogenomics Database. Database (Oxford) 2012:bar065.

Davis AP, Grondin CJ, Lennon-Hopkins K, Saraceni-Richards C, Sciaky D, King BL, et al. 2014. The Comparative Toxicogenomics Database's 10th year anniversary: update 2015 Nucleic Acids Res.

DECIPHER. 2015. Available: https://decipher.sanger.ac.uk [accessed 10 October 2015].

Entrez-Gene (NCBI Entrez-Gene). 2015. Available: http://www.ncbi.nlm.nih.gov/entrez [accessed 10 October 2015].

Kohler S, Doelken SC, Mungall CJ, Bauer S, Firth HV, Bailleul-Forestier I, et al. 2014. The Human Phenotype Ontology project: linking molecular biology and disease through phenotype data. Nucleic Acids Res 42:D966-974.

MeSH (NLM Medical Subject Headings). 2015. Available: http://www.nlm.nih.gov/mesh [accessed 10 October 2015].

Monarch (The Monarch Initiative). 2015. Available: http://monarchinitiative.org [accessed 10 October 2015].

obophenotype. 2016. Available: https://github.com/obophenotype [accessed 10 October 2015].

OMIM (Online Mendelian Inheritance in Man). 2015. Available: http://www.ncbi.nlm.nih.gov/omim [accessed 10 October 2015].

orphanet. 2015. Available: http://www.orphadata.org/cgi-bin/inc/ordo_orphanet.inc.php [accessed 10 October 2015].

RedCap (Research Electronic Data Capture system). 2016. Available: https://pedsredcap.uthsc.edu/redcap/ [accessed 1 February 2016].

Taxonomy (NCBI Taxonomy). 2015. Available: http://www.ncbi.nlm.nih.gov/taxonomy [accessed 10 October 2015].
